# Determination of the optimal ejaculate concentration of a buffalo bull for successful semen cryopreservation

**DOI:** 10.3389/fvets.2025.1594298

**Published:** 2025-07-15

**Authors:** Krishna Bansal, Dinesh Jhamb, Usha Yadav, Mamta Meel, Akanksha Gupta, Renu Bala, Meenakshi Virmani, Meeti Punetha, Dharmendra Kumar, Pradeep Kumar

**Affiliations:** ^1^Animal Physiology and Reproduction Division, ICAR-Central Institute for Research on Buffaloes, Hisar, Haryana, India; ^2^Department of Veterinary Gynaecology and Obstetrics, College of Veterinary and Animal Science, Rajasthan University of Veterinary and Animal Sciences, Bikaner, Rajasthan, India; ^3^Division of Animal Reproduction, ICAR-Indian Veterinary Research Institute, Bareilly, Uttar Pradesh, India; ^4^Department of Dairy Cattle Physiology, ICAR-National Dairy Research Institute, Karnal, India; ^5^Department of Veterinary Physiology and Biochemistry, Lala Lajpat Rai University of Veterinary and Animal Sciences, Hisar, Haryana, India

**Keywords:** buffalo, ejaculate, sperm, rejection rate, semen

## Abstract

For cattle bulls, only ejaculates with a sperm concentration of 500 million/mL or higher are selected for sperm cryopreservation. There is no established ejaculate’s minimum sperm concentration threshold for buffalo semen cryopreservation. Therefore, the first objective of the study was to determine the percentage of ejaculates of buffalo bulls having concentrations lower than 500 million/mL, the percentage of ejaculates suitable for cryopreservation across different concentrations of donated ejaculates, and to estimate the impact of ejaculate concentrations on the production of semen doses. The second objective was to evaluate the post-thaw sperm quality of ejaculates that initially met the criteria for cryopreservation. After analyzing 5,347 ejaculates from 31 buffalo bulls, it was found that 9.96% of the buffalo semen ejaculates had a sperm concentration of less than 500 million/mL. Of these, 48% qualified the minimum criteria, mainly based on sperm motility. The maximum semen doses (8.79%) were produced by the ejaculates with a sperm concentration between 1,101 and 1,201 million/mL. For the second objective of the study, the ejaculates were categorized into four groups: <300, 300–400, >400- < 500, and ≥ 500 million sperm/mL, diluted to a final concentration of 80 million sperm/mL and cryopreserved. The frozen–thawed sperm of different groups were evaluated by a computer-assisted sperm analyser (CASA) and a flow cytometer. Remarkably, it was found that sperm motility, integrity of plasma and acrosome membrane, and mitochondrial membrane potential (MMP) significantly decreased (*p* < 0.05) when the ejaculates of category <300 were cryopreserved, despite being qualified in subjective motility analysis compared to the other categories. Except for the category <300, in all the other three categories, there was no significant difference (*p* > 0.05) in the percentage of spermatozoa in MMP. The highest and lowest percentages of spermatozoa with high mitochondrial superoxide were found in categories <300 and ≥500, respectively. In conclusion, buffalo semen ejaculates with a concentration greater than 300 million sperm/mL should be considered for cryopreservation.

## 1 Introduction

The buffalo population in India is 109.9 million, which is 56.5% of the world buffalo population (1). To increase buffalo milk production, a continuous breeding programme is ongoing through artificial insemination using cryopreserved semen of elite buffalo bulls. In India, there is an insufficient number of elite breeding bulls (only 2,294) to cover all the breeding buffalo (2). Therefore, there is a need to develop different strategies for producing frozen semen doses from the existing breeding buffalo bulls. In this context, it is important to emphasize that buffalo and cattle exhibit significant differences in their genetic composition, specifically in their chromosome pairs, 50 in buffalo and 60 in cattle. Hence, the physiology of male reproduction of cattle and buffalo bulls differs in many aspects. The testis size of buffalo bulls is smaller than that of cattle bulls. The daily sperm production of buffalo bulls is also lower than that of cattle bulls. The average sperm concentration (900 vs. 1,200 million/mL) of the ejaculate of buffalo bulls is lower than that of cattle bulls (3, 4). Compared to European breeds, daily sperm output in Murrah bulls was nearly 45% lower, presumably due to their nearly 40% lower scrotal circumference than Holstein bulls of the same age (5). Despite the scientific facts, there is no separate guideline and recommendation for producing frozen semen doses of buffalo bulls. For cattle bulls, there are some guidelines stating that “the ejaculate having a sperm concentration lower than 500 million sperm per mL shall be rejected for use in the production of frozen semen doses” (6, 7). There is no report on the minimum ejaculate concentration of a buffalo bull suitable for cryopreservation. Therefore, the study’s first objective was to analyze the data collected from 2018 to 2022 in our laboratory to assess the percentages of donated ejaculate concentration lower than 500 million sperm per millilitre and evaluate the success rates of cryopreserving the donated ejaculates compared to ejaculates with more than 500 million sperm/mL. One of the most crucial aspects of fertile semen is sperm motility, but motility alone may not be a reliable indicator of bull fertility. Therefore, the second objective of this study was to evaluate, in more depth, the post-thaw semen quality of the ejaculates that had initially been assessed based on subjective analysis of sperm motility, using Computer-assisted sperm analysis (CASA) and a flow cytometer. This is because traditional microscopic-based quality control assessments alone may not provide a comprehensive evaluation of semen quality and the ability to predict bull fertility.

## 2 Materials and methods

### 2.1 Ethics statement

The Institute Research Ethics Committee has confirmed that no ethical approval is required for the study (IAEC-CIRB/104).

### 2.2 Experimental location and design

The research was conducted at the Semen Freezing Laboratory of the Division of Animal Physiology and Reproduction, ICAR-Central Institute for Research on Buffaloes, Hisar (latitude 29.1852288, longitude 75.7002471) between March 2022 and October 2022. In the study, the data of semen collection during the period 2018–2022 were also analyzed. For the first objective of the study, the 5,650 semen ejaculates collected from 31 bulls during the period between 2018 and 2022 were analyzed. The percentages of qualified ejaculates per bull from 31 Murrah buffalo bulls were calculated. Furthermore, the percentages of donated ejaculates with sperm concentrations of less than 500 million/mL out of the total 5,650 ejaculates contributed by the 31 bulls were determined. In addition, 702,741 semen doses were produced during the period, contributed by varying ejaculate concentration ranges differing by 100 million/mL. This in-depth study helped us determine the percentages of qualified ejaculates at various sperm concentrations, offering valuable insights into the reproductive potential of male buffaloes.

### 2.3 Conducting data mining to analyze the semen ejaculate concentrations donated by buffaloes

For objective 1, we evaluated the performance of Murrah buffalo bulls in tropical conditions by analyzing semen collected in our laboratory from 2018 to 2022. The assessment included analyzing the percentage of qualified semen ejaculates with varying sperm concentrations, determining the proportion of ejaculates meeting the minimum recommended criteria for sperm cryopreservation from specific buffalo bulls, assessing the percentage of ejaculates with sperm concentrations below 500 million/mL from specific bulls, and quantifying the semen doses derived from ejaculates with differing concentrations.

### 2.4 Semen collection

**Figure 1:**
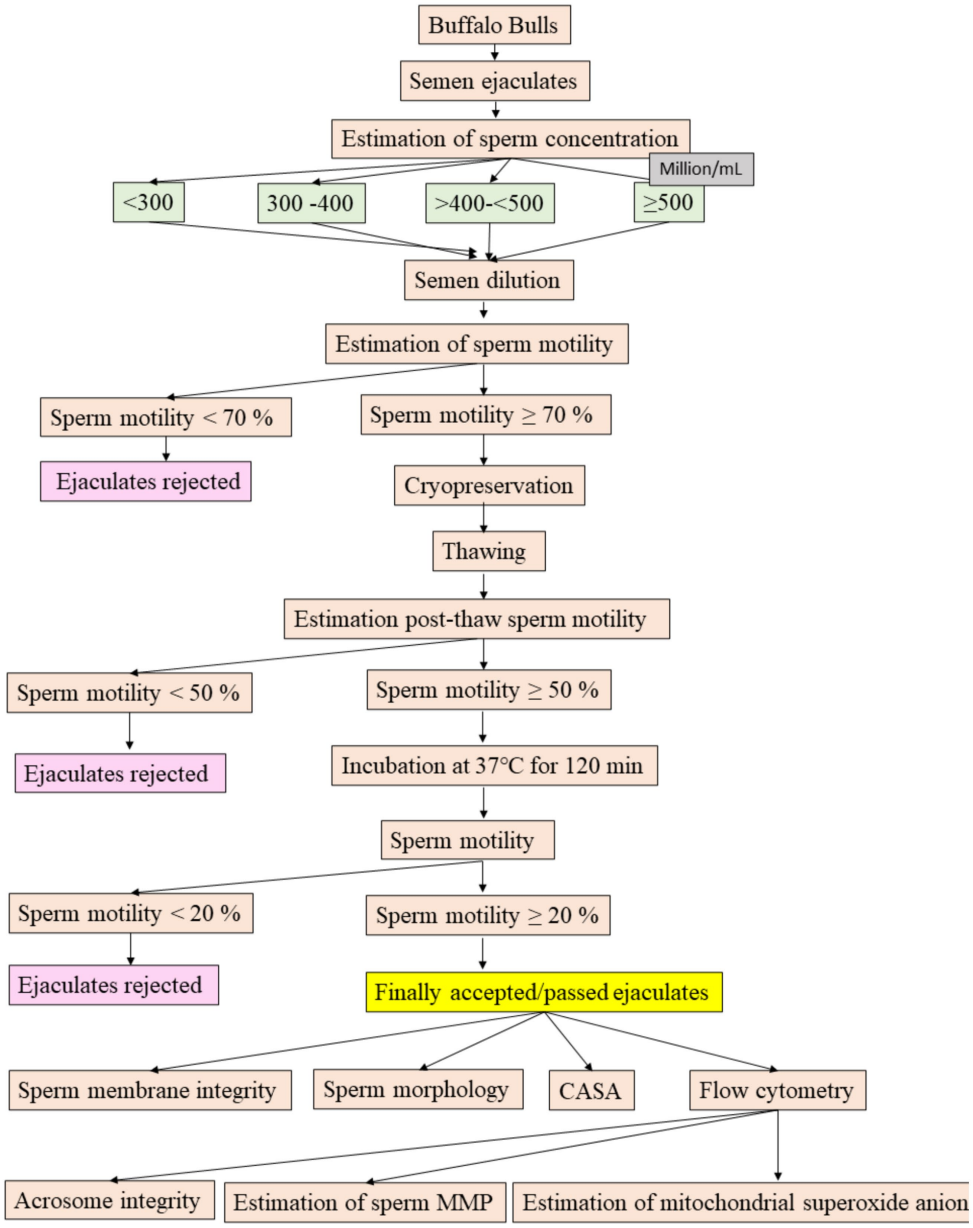
Experimental design.

The flow diagram illustrating the experimental design for objective 2 can be found in Figure 1. The semen was carefully collected from the buffalo bulls using an artificial vagina in the early morning before feeding. Each bull contributed two ejaculates on the collection day, with a 30-min interval between the two collections.

### 2.5 Estimation of sperm concentration

The sperm concentration (million/mL) in the freshly collected semen was measured using an AccuCell bovine photometer (IMV, L‘Aigla, France) at a 530 nm wavelength. To obtain the measurement, semen samples (40 μL) were diluted with normal saline 0.9% w/v (3,960 μL) using an automatic diluter. The absorbance of the diluted semen was then determined with the AccuCell bovine photometer to calculate the sperm concentration (million/mL) of the ejaculate. Based on sperm concentration, the ejaculates were divided into four categories: <300, 300–400, >400- < 500, and ≥ 500 million sperm/mL. The classification of ejaculates based on the recommendations and guidelines of the bovine semen production guideline, in which it is mentioned that the ejaculate concentration less than 500 million/mL shall not be used for cryopreservation (6, 7). Based on the recommendation, we categorised the bubaline (buffalo) ejaculates as less than 500 million/mL and more than 500 million/mL ejaculates. Further, less than 500 million/ml ejaculates were subcategorised to determine how much less than 500 million/ml ejaculates could be utilized for semen production in buffaloes.

### 2.6 Cryopreservation

The initial dilution of semen involved mixing an equal quantity of diluent and maintaining it in a water bath at 32°C (8). Upon determining the sperm concentration, the diluent (tris, fructose, citric acid, egg yolk, streptomycin, penicillin and glycerol) was added to the pre-diluted semen to achieve a final concentration of 80 million sperm/mL. Subsequently, sperm motility was evaluated by placing a small drop of the diluted semen on a clean, grease-free warm slide (37°C), covering it with a slip, and examining it under a phase contrast microscope at 200x magnification (8). For cryopreservation consideration, the ejaculates were required to exhibit a minimum of 70% sperm motility as per the minimum standard for the production of bovine frozen semen of the Indian Government. The extended semen was loaded into 0.25 mL plastic straws (IMV, L’Aigle, France), and gradually cooled to 4°C. It was then equilibrated for 4 h in a cold cabinet and subsequently frozen using a programmable biological freezer (Mini Digi-cool, IMV Technologies, L’Aigle, France) as previously described in our laboratory (9). Each semen sample was cooled at a precise rate of −5°C/min from 4°C to −10°C. Between −10°C and −100°C, the freezing rate was −40°C/min, and then from −100°C to −140°C, the rate was −20°C/min. After reaching −140°C, the semen straws were immediately immersed in liquid nitrogen at −196°C for storage.

### 2.7 Post-thaw sperm motility

Upon completion of a 24-h cryopreservation period, the semen straws were thawed at 37°C for 30 s, following which an immediate estimation of sperm motility was performed. Subsequently, ejaculates exhibiting a minimum of 50% sperm motility were selected for further incubation at the same temperature. Sperm motility was assessed at 30-min intervals throughout the 120-min incubation period. Following the incubation period, ejaculates having more than 20% sperm motility were classified as passed/qualified ejaculates; those failing to meet this criterion were considered rejected ejaculates (10). The percentage of passed and failed ejaculates for all categories was then computed. Additionally, passed ejaculates from all categories were subjected to evaluations encompassing sperm morphology, plasma membrane integrity, CASA parameters, acrosome integrity, mitochondrial membrane potential, and mitochondrial superoxide anion status. This assessment was conducted to evaluate vital parameters beyond sperm motility for suitability in artificial insemination.

### 2.8 Sperm morphology

Eosin-nigrosin staining was carried out on cryopreserved semen samples to assess sperm morphology, following the methodology outlined by Kumar et al. (9). Upon thawing each semen straw at 37°C, a small drop of the thawed semen was deposited onto a clean, grease-free prewarmed slide. A thin smear was meticulously prepared by utilizing a spreader slide at a 30-degree angle to distribute the semen suspension over the slide length uniformly. Then it was fixed through air drying. About 200 spermatozoa from each sample were scrutinized across diverse fields at 100X objective under a phase-contrast microscope to ascertain the percentage of abnormal spermatozoa manifesting characteristics such as bent tails, coiling of tails over the midpiece, proximal protoplasmic defects, and eccentric thickening of the acrosome.

### 2.9 Sperm plasma membrane integrity

To evaluate the functional integrity of the sperm plasma membrane, a hypo-osmotic swelling test (HOST) was conducted in accordance with the methods outlined by Jeyendran et al. (11). Specifically, 500 μL of a hypo-osmotic solution (consisting of 0.735 g of sodium citrate dihydrate and 1.351 g of fructose in 100 mL of double-distilled water, resulting in a 100 mOsm/L solution) was combined with 50 μL of frozen–thawed semen. In parallel, a control group was prepared by mixing 500 μL of PBS with 50 μL of frozen–thawed semen, followed by incubation at 37°C for 60 min. Following incubation, 40 μL of 2% eosin solution was introduced to enhance visibility. Subsequently, a drop of diluted semen was placed onto a clean, sterilized, and dry glass slide and covered with a coverslip. Morphological assessment was performed by enumerating 200 spermatozoa in distinct fields at 100x magnification under a phase-contrast microscope to determine the percentage of spermatozoa exhibiting HOST-reactivity, characterized by the presence of coiled tails. The percentage of coiled-tail spermatozoa in the PBS group was subtracted from that of the HOST to ascertain the true HOST-reactive sperm. Spermatozoa displaying coiled tails after incubation were deemed to possess an intact plasma membrane.

### 2.10 Sperm motility and kinematic parameters

The sperm kinematics and motility parameters were evaluated utilizing the computer-assisted sperm analyzer (CASA, Hamilton Thorne IVOS II). Preceding analysis through CASA, the semen specimen was diluted with pre-warmed Tris buffer to achieve a sperm concentration of approximately 40 million spermatozoa per millilitre. Subsequently, a volume of two microliters of the aforementioned diluted semen sample was introduced to a pre-warmed (37°C) eight-chamber Leja slide with a depth of 20 μm to assess sperm kinematics and motility characteristics. Variables such as total sperm motility (TM %), progressive sperm motility (PM %), straight linear velocity (VSL, μm/s), average path velocity (VAP, μm/s), curvilinear velocity (VCL, μm/s), amplitude of lateral head displacement (ALH, μm), beat cross frequency (BCF, Hz), straightness (STR, %), linearity (LIN, %), and Wobble (WB %) were recorded. The settings of the CASA software encompassed a temperature of 37°C, frame rate of 60 Hz, acquisition of 30 frames, and specific parameters including minimum contrast of 35, minimum cell size of 5 pixels, cell size of 9 pixels, cell intensity of 110 pixels, as well as the categorization of progressive cells based on specific velocity and straightness cut-off values (VAP cut-off = 50 μm/s, STR cut-off = 70%).

### 2.11 Estimation of sperm mitochondrial superoxide anion status

To assess the functionality of sperm mitochondria in ejaculates with varying concentrations, the excess superoxide anions produced by mitochondria were detected using MitoSox Red. Compromised mitochondria primarily produce excess superoxide anions during ATP production. MitoSox Red, a specific fluorescent mitochondrial superoxide indicator, is only oxidized by superoxide, not other free radicals. The oxidized product becomes highly fluorescent after binding to nucleic acid. The mitochondrial superoxide anion status in sperm cells was quantified using MitoSox Red (Thermo Fisher Scientific USA, #M36008) and analyzed via flow cytometry, as Patil et al. (12) described. In brief, washed frozen–thawed sperm were mixed with a working solution of MitoSox (5 μM) to achieve a final dye concentration of 0.125 μM and incubated at 37°C for 15 min in the dark. After incubation, the sperm were washed twice to remove excess dye in suspension and analyzed using a flow cytometer (CytoFLEX, Beckman Coulter-Life Sciences). Excitation was performed using a blue laser (488 nm), and detection was performed with a 585/42 BP (FL-2 channel: PE). A total of 20,000 events were recorded for each sample.

To detect the sperm cells, the P1 population was obtained based on the FSC-A vs. FSC-H dot plot, excluding doublets and debris from the main population using a polygon gate. The obtained P1 population was then gated based on the FSC-A vs. SCC-A dot plot to obtain the P2 population. The P2 population was then further analyzed using auto-line segmentation on a histogram. The gated population was classified into MitoSOX-positive and MitoSOX-negative based on running the sample without the dye as a control. A MitoSoX-positive sperm population indicated compromised sperm mitochondria and excess superoxide anion production. All data was collected and analyzed using CytExpert software (v.2.3) (CytoFLEX, Beckman Coulter-Life Sciences).

### 2.12 Estimation of sperm mitochondrial membrane potential (MMP)

To determine the proportion of sperm with high mitochondrial membrane potential in ejaculates of varying concentrations, JC-1 fluorescent dye was employed as an indicator of mitochondrial health. The JC-1 dye demonstrates potential-dependent accumulation in mitochondria, manifesting a shift in fluorescence emission from green (529 nm) to red (590 nm). Mitochondria harbouring red JC-1 aggregates, indicative of high mitochondrial membrane potential in healthy cells, are discernible in the FL-2 channel (PE), while apoptotic cells with low membrane potential display green JC-1 monomers identifiable in the FL-1 channel (FITC). Measurement of sperm mitochondrial membrane potential was conducted utilizing JC-1 (Thermo Fisher Scientific USA, # T3168). The processed frozen–thawed semen was combined with a working JC-1 (1.5 mM) solution to yield a final concentration of 2 μM JC-1, followed by a 15-min incubation at 37°C in darkness. Subsequently, the sample was subjected to two washes and analyzed using a flow cytometer (CytoFLEX, Beckman Coulter-Life Sciences), with excitation achieved by a blue laser (488 nm) and detection using FL-1 channel (FITC) and FL-2 channel (PE). Each sample recorded a total of 20,000 events. To ensure data precision, the P1 population was isolated from the FSC-A vs. FSC-H dot plot by excluding doublets and debris by implementing a polygon gate. The derived P1 population was then gated under the FSC-A vs. SCC-A dot plot to procure the P2 population. This P2 population was detected in FL1 (FITC) vs. FL2 (PE), segregating two distinct populations. This gated population was classified into two cohorts, namely JC-1 aggregates/High mitochondrial membrane potential (High MMP) and JC-1 monomers/low mitochondrial membrane potential (Low MMP). All data were amassed and analyzed employing CytExpert software (v.2.3) (CytoFLEX, Beckman Coulter-Life Sciences).

### 2.13 Estimation of acrosomal integrity

To identify the percentage of live sperm with intact acrosomes in different categories of ejaculates, *Pisum sativum* agglutinin (PSA) and propidium iodide (PI) fluorescent dyes were used. The PSA cannot permeate the intact outer acrosomal membrane, but in the damaged acrosome membrane, it binds to mannose and agalactose moieties of the acrosomal matrix. Similarly, PI gives red fluorescence in dead sperm. The washed, frozen–thawed sperm were incubated at 37°C for 10 min, and then 5 μL PI was added. The samples were analyzed using a flow cytometer and classified into four groups: live sperm with intact acrosome, live sperm with damaged acrosome, dead sperm with intact acrosome, and dead sperm with damaged acrosome.

### 2.14 Statistical analysis

The statistical analysis was conducted utilizing SPSS software version 22 (IBM Corporation, Armonk, NY, USA) to evaluate differences among groups. The distribution of semen variables data was meticulously assessed using the Shapiro–Wilk test to check for normality. In cases where the distribution was deemed abnormal, we applied an arcsine transformation to the data to ensure a normalized distribution before analysis. Furthermore, we ensured the comparability of variances among groups by conducting Levene’s test using the SPSS statistical package. Subsequently, we utilized one-way ANOVA to analyze the data and employed the Duncan multiple range tests (DMRT) to compare means. To establish statistical significance, the threshold was set at the 0.05 probability level. The results are presented as mean values alongside the standard error (SE).

## 3 Results

### 3.1 Percentages of buffalo semen ejaculates with different sperm concentrations not rejected after cryopreservation

**Figure 2:**
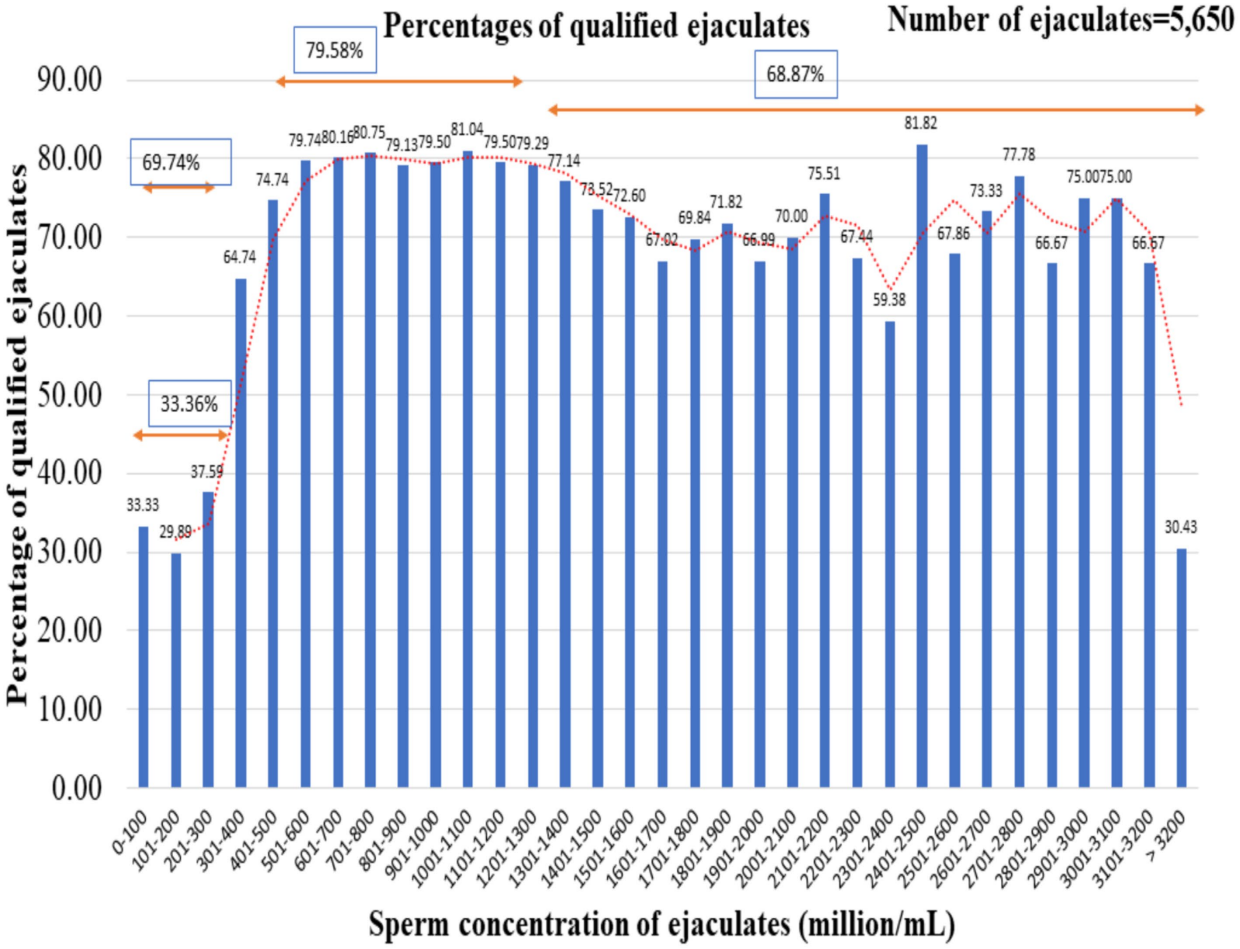
Percentages of ejaculates that met the essential criteria for successful cryopreservation. The total number of ejaculates was 5,650.

The percentage of ejaculates qualified based on initial sperm motility (70%), post-thaw sperm motility (50%), and sperm motility after incubation at 37°C for 120 min (20%) was calculated from 5,650 ejaculates collected by buffalo bulls in our laboratory between 2018 and 2022 (Figure 2). It was observed that 33.36% of the ejaculates qualified for the minimum criteria of ejaculates having a concentration of less than 300 million sperm/mL. The ejaculates with concentrations ranging from 301 to 400 million sperm/mL and 401 to 500 million sperm/mL accounted for 64.74 and 74.74% of the cryopreserved ejaculates, respectively. Further, it was observed that the highest percentage (79.58%) of passed ejaculates were ejaculated with a concentration range of between 501 and 1,300 million sperm/mL. The noteworthy finding of the observation was that the percentage of qualified ejaculates declined as the concentration of ejaculates increased from 1,500 million sperm/mL.

### 3.2 Percentage of ejaculates of a particular buffalo bull qualified the minimum recommended criteria for sperm cryopreservation

**Figure 3:**
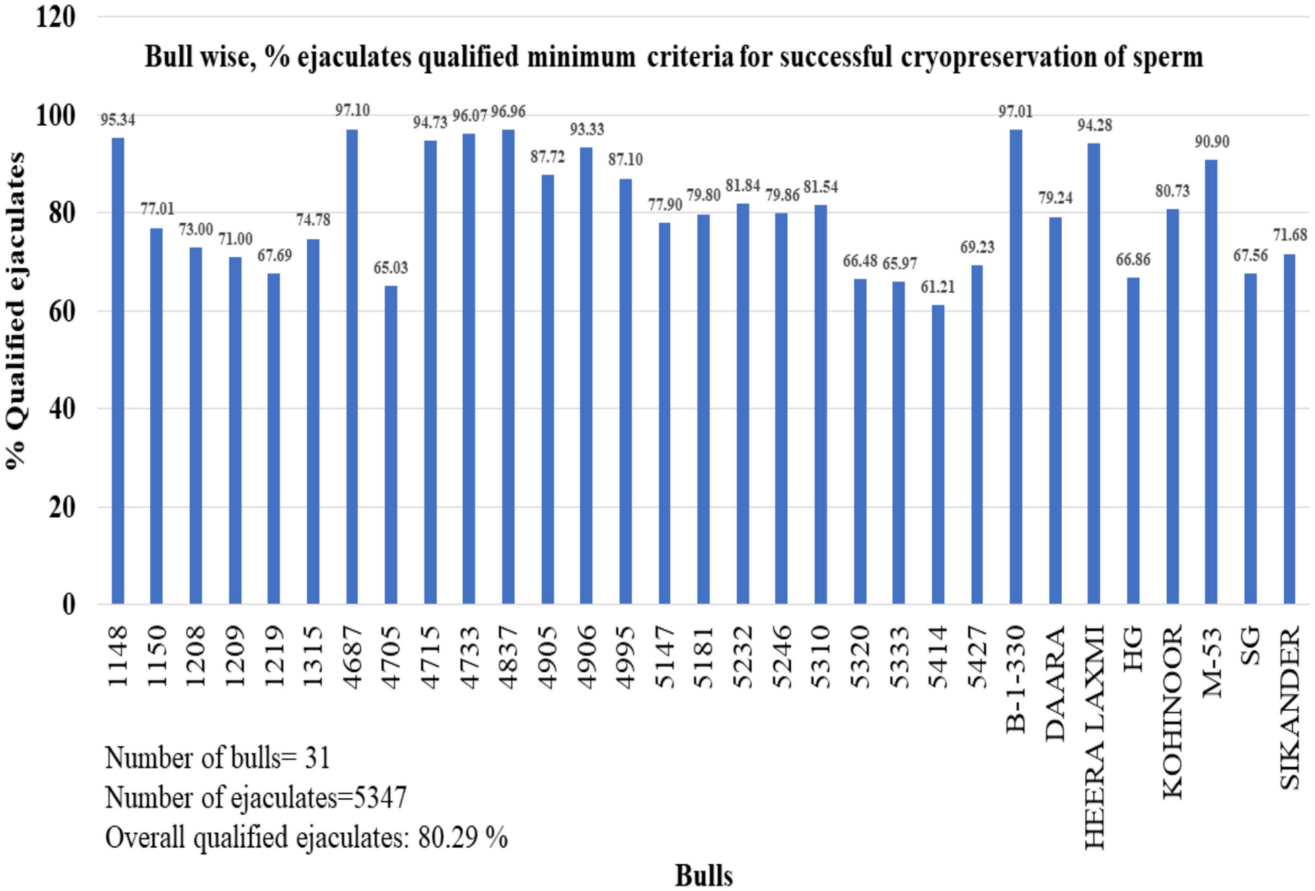
Percentage of ejaculates that met minimum qualifying criteria of a particular buffalo bull. The bulls not included in the study that have not completed at least 30 ejaculates. A total of 5,347 ejaculates of 31 bulls were included in the study.

To analyze the percentage of qualified ejaculates in buffalo bulls, we examined the data from 31 bulls. The range of qualified ejaculates was 60 to 97%, with an overall average of 80.29% (Figure 3).

### 3.3 Percentage of ejaculates of a bull buffalo with sperm concentration below 500 million/mL

**Figure 4:**
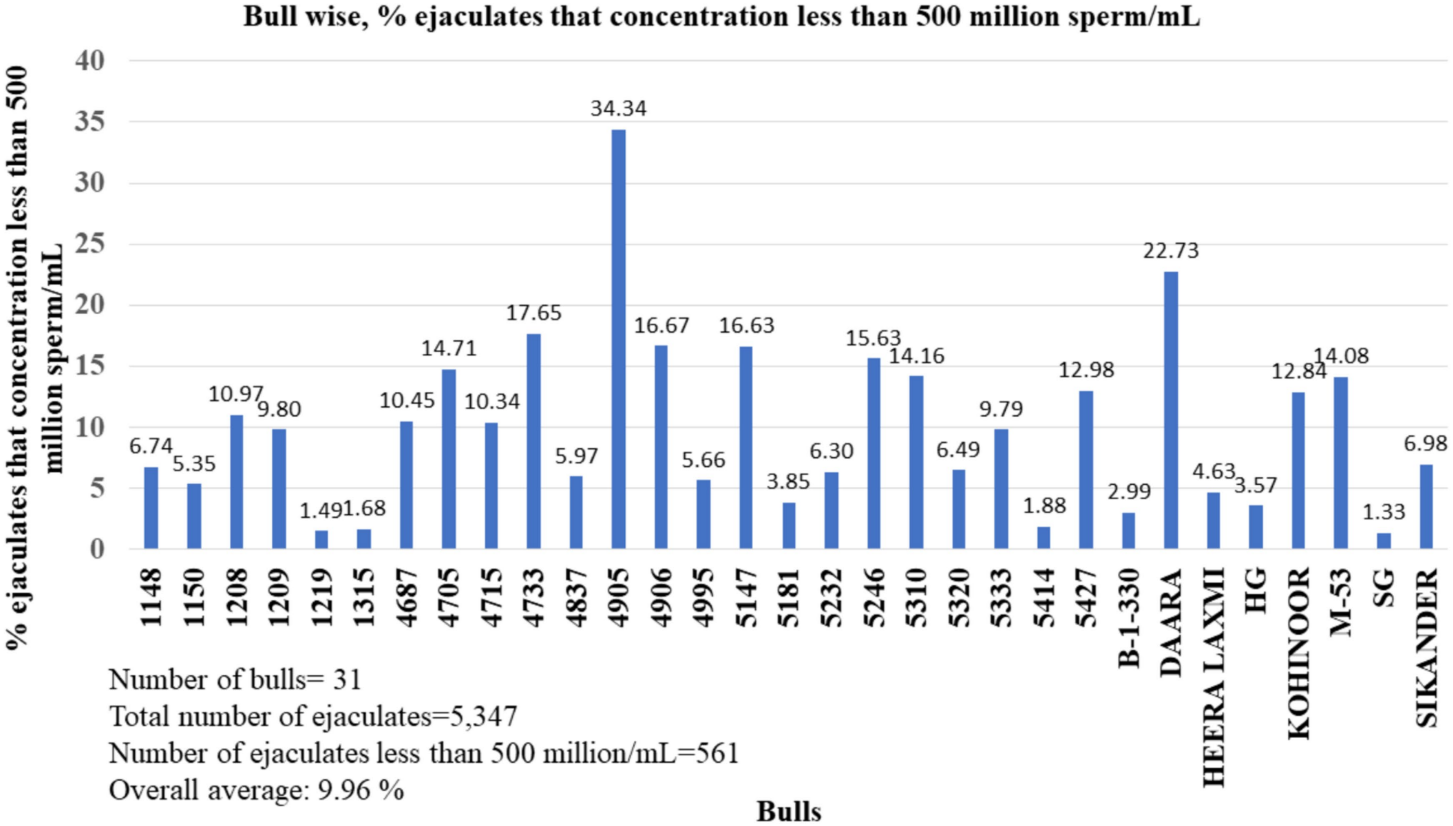
Percentages of ejaculates donated by buffalo bulls that had a concentration less than 500 million sperm/mL.

The ejaculate concentration of particular buffalo bulls also varied depending on several factors. Every buffalo breeding bull many times donates ejaculates with a sperm concentration of less than 500 million sperm/mL. In the bull-wise study, the percentage of the ejaculate’s concentration was less than 500 million/mL, ranging from 1.33 to 34.34% (Figure 4). The average percentage of buffalo semen ejaculated at a concentration of less than 500 million/mL was 9.96%.

### 3.4 Semen doses produced by the buffalo ejaculates of different concentrations

**Figure 5:**
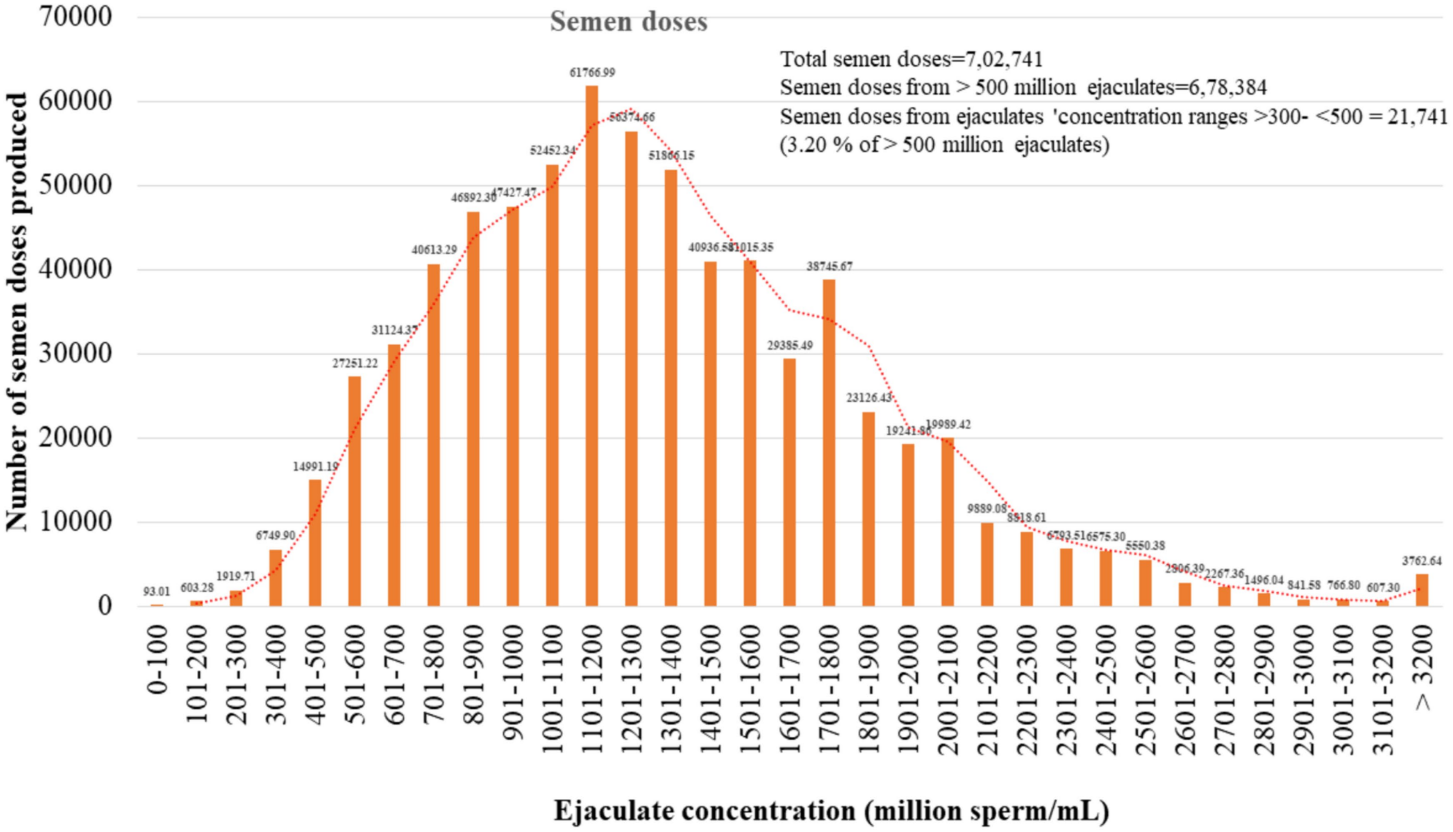
Estimation of semen doses prepared by ejaculates that met minimum qualifying criteria. The total semen doses produced during the last 5 years in our laboratories were used for the study. The total semen doses produced were 7,02,741.

Finally, the contribution of ejaculates in terms of semen dose production was estimated from our records for the period 2018–2022. The semen doses produced by different concentration ranges of ejaculates are in Figure 5. Out of 702,741 semen doses, 6,749 (0.27%), 14,991 (2.13%), and 27,251 (3.8%) semen doses were produced by the ejaculates with sperm concentrations of 301–400, 401–500, and 501–600 million sperm/mL, respectively.

### 3.5 Impact of varying ejaculate concentrations on sperm motility, velocity, BCF, ALH, STR, LIN, and WOB

The post-thaw sperm motility and kinematics parameters of qualified ejaculates from different categories were assessed using CASA (Table 1). The total and progressive sperm motility of ejaculates of category <300 million sperm/mL was lower (*p* < 0.05) than that of category ≥500 million sperm/mL. The sperm motility of the rest of the two categories did not differ significantly (*p* > 0.05) from the lowest and highest sperm motility-bearing categories. The different sperm velocities (VCL, VAP, and VSL) of all four categories were in the reported ranges and did not differ (*p* > 0.05). Similarly, BCF and ALH of sperm from all four categories were comparable (*p* > 0.05). The STR, LIN, and WOB of sperm in all the categories were equal (*p* > 0.05).

**Table 1:** Sperm motility, velocity parameters, membrane integrity and abnormal sperm in different groups of ejaculates.

Parameters	Sperm concentration of ejaculates (million/mL)			
	<300	300–400	>400– < 500	≥500
TM (%)	37.91 ± 2.10^b^	45.11 ± 2.37^ab^	48.46 ± 2.88^ab^	56.75 ± 2.68^a^
PM (%)	31.91 ± 2.08^b^	39.57 ± 2.27^ab^	40.29 ± 2.59^ab^	46.64 ± 3.12^a^
VCL (μm/s)	174.86 ± 8.37	167.49 ± 10.32	168.38 ± 6.22	161.79 ± 6.72
VAP (μm/s)	110.50 ± 5.25	103.12 ± 5.12	102.82 ± 3.65	100.49 ± 4.35
VSL (μm/s)	98.36 ± 4.69	86.57 ± 3.91	86.61 ± 3.34	87.63 ± 4.11
BCF (Hz)	30.30 ± 0.31	29.39 ± 0.89	30.97 ± 0.42	30.83 ± 0.49
ALH (μm)	7.08 ± 0.30	7.10 ± 0.49	6.96 ± 0.23	6.60 ± 0.22
STR (%)	86.83 ± 0.58	83.01 ± 1.35	85.65 ± 0.70	86.04 ± 0.76
LIN (%)	57.11 ± 1.10	53.46 ± 1.78	54.65 ± 0.89	56.25 ± 1.35
WOB (%)	63.56 ± 0.85	62.73 ± 1.22	62.43 ± 0.64	63.91 ± 1.17
HOST reactive (%)	35.00 ± 2.99^c^	40.83 ± 1.81^bc^	41.80 ± 1.16^ab^	43.45 ± 3.73^a^
Abnormal Sperm (%)	18.04 ± 1.98^b^	14.07 ± 2.59a^a^	12.85 ± 3.37^a^	12.43 ± 2.70^a^

The ejaculates were categorized into four groups based on sperm concentration (million/mL) of ejaculate: <300, 300–400, >400– < 500, and ≥500. Results are expressed in mean ± standard error. Different superscripts ^a–c^ indicate statistical differences (*p* ≤ 0.05) among different groups in each row, Total sperm motility (TM), Progressive sperm motility (PM), average path velocity (VAP), curve linear velocity (VCL), straight linear velocity (VSL), amplitude of lateral head displacement (ALH), beat cross frequency (BCF), straightness (STR), linearity (LIN) and wobble (WOB). Number of ejaculates/each group = 10.

### 3.6 Low-concentrated ejaculates adversely affect membrane integrity and morphology of post-thaw sperm

The sperm plasma membrane integrity of all four categories was evaluated by HOST to determine the impact of low-concentrated ejaculates on the sperm membrane (Table 1). It was found that higher (*p* < 0.05) plasma membrane integrity was in the categories of ejaculates having a concentration ≥500 million sperm/mL than in the category of ejaculates having a sperm concentration <300 million/mL. However, the plasma membrane integrity of the other two categories (300–400 and >400– < 500) were between the two categories and lies under the recommended level. The sperm abnormalities of category <300 were higher (*p* < 0.05) than those of the rest categories (Table 1). There were no significant differences (*p* > 0.05) in sperm abnormalities in the other three categories. However, the percentage of sperm abnormalities in all four categories was under the recommended level.

### 3.7 Percentage of post-thaw sperm with intact acrosomes decreases as ejaculate’s concentration decreases

**Figure 6:**
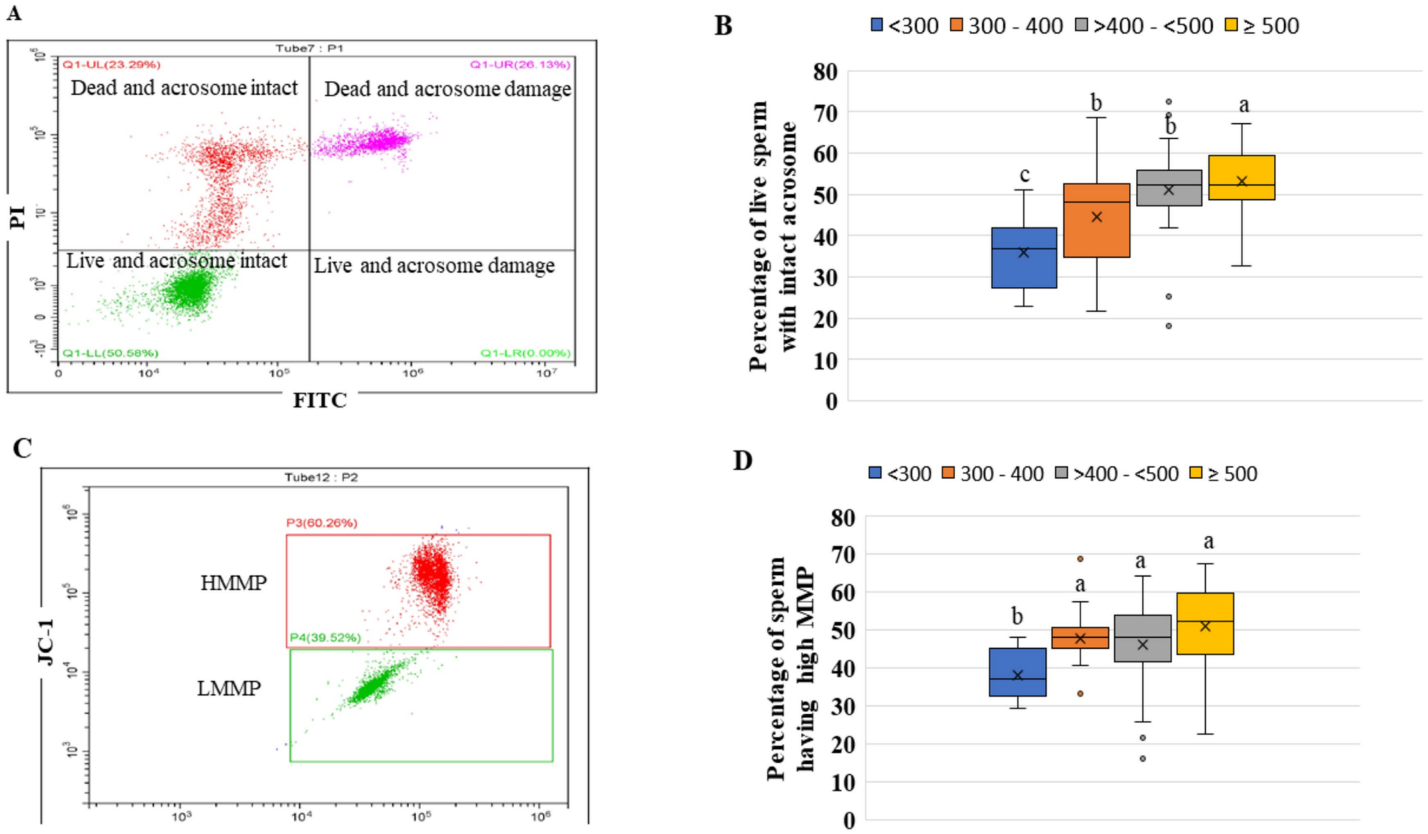
**(A)** Acrosome integrity of frozen-thaw sperm: The selected sperm cells population were gated and classified into four windows: lower left window -live sperm with intact acrosome (live intact), lower right window: live sperm with damaged acrosome (live damage), upper left window: dead sperm with intact acrosome (dead intact) and upper right window- dead sperm with damaged sperm (dead damage), **(B)** the percentage live sperm with intact acrosome in the four groups of ejaculates, **(C)** showing two gated populations viz., HMMP (red color) and LMMP (low mitochondrial membrane potential, green color), **(D)** Percentage of HMMP sperm in the four groups of the ejaculates. The ejaculates were categorized into four groups based on sperm concentration (million/mL) of ejaculate: <300, 300–400, >400- < 500, and ≥500. Different superscripts differ significantly (*p* < 0.05). Number of ejaculates/each group = 10.

The intactness of the acrosome in sperm is a crucial criterion for the use of artificial insemination purposes. The highest (*p* < 0.05) number of live spermatozoa with intact acrosome was found in categories ≥500 (Figures 6A,B). The lowest (*p* < 0.05) number of live spermatozoa with intact acrosome was in the category <300. Except for category <300, the percentage of live spermatozoa with intact acrosomes was within acceptable limits in all categories.

### 3.8 Mitochondrial membrane potential (MMP) of frozen–thawed sperm from extremely low-concentrated ejaculates is compromised

The high mitochondrial membrane potential in sperm is essential for normal mitochondrial function, ATP production, traveling in the female reproductive tract, and penetration of spermatozoa into oocytes. Except for the category <300, in all the other three categories, there was no significant difference (*p* > 0.05) in the percentage of spermatozoa with high MMP (Figures 6C,D).

### 3.9 Low concentrations of ejaculates significantly elevate mitochondrial superoxide anion production in frozen–thawed sperm

**Figure 7:**
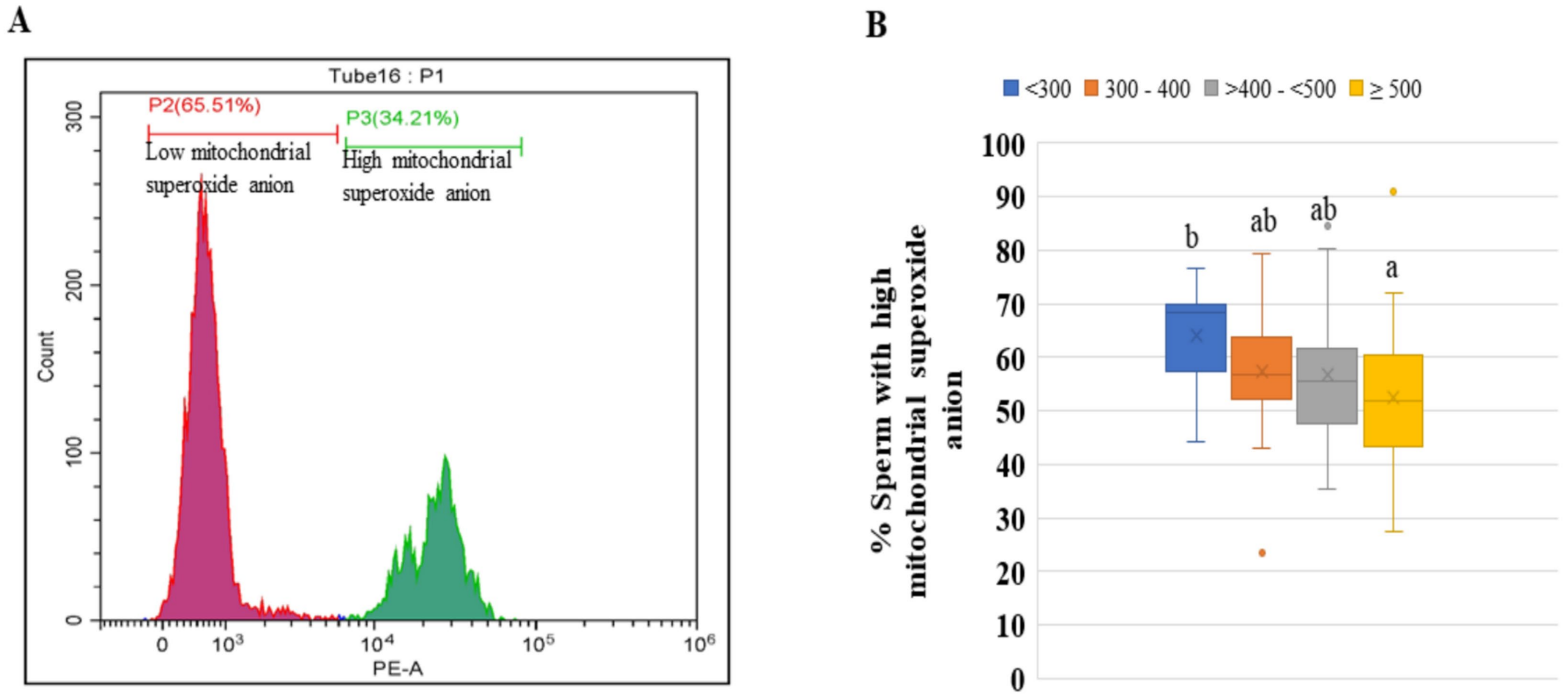
Percentages of sperm cell populations with high concentrations of superoxide anion in the four groups of the ejaculates. **(A)** Histogram showing two gated populations viz., sperm cell population having a low concentration of superoxide anion (red color) and sperm cell population having a high concentration of superoxide anion (green color). **(B)** The percentages of sperm with high mitochondrial superoxide anion in the different groups. The ejaculates were categorized into four groups based on sperm concentration (million/mL) of ejaculate: <300, 300–400, >400– < 500, and ≥500. Different superscripts differ significantly (*p* < 0.05). Number of ejaculates/each group = 10.

The compromised or damaged sperm mitochondria produce an excess amount of superoxide during ATP production, which is harmful to sperm functionality and fertility. Hence, using the specific fluorescent dye MitoSOX, which selectively measures the superoxide anion produced by mitochondria, the number of compromised sperm with high superoxide production was estimated in all four categories. The greater percentage of spermatozoa with high mitochondrial superoxide was found in category <300 compared to the other three categories (Figures 7A,B).

## 4 Discussion

Although both buffalo and cattle bulls are classified as large ruminants, their sperm characteristics vary due to different genetic makeup. The concentration of spermatozoa in an ejaculate is species-specific. The mean sperm concentration of buffalo was recorded as 977.11 million/ml, which ranges from 220 to 1,740 million/ml in Murrah bull semen (4, 5, 13), which is lower than that of cattle. The collection of a ‘high-quality’ ejaculate containing high sperm concentration and wave motion is no guarantee of successful semen cryopreservation. In humans, oligozoospermic is defined as donating most ejaculates with a sperm concentration of less than 15 million sperm/mL (14). This may be due to pathological reasons. But, the bull selection procedure for frozen semen production is strict. During the selection process, those bulls that have consistently shown poor libido, watery ejaculates, and a high ejaculate rejection rate are not kept for semen production despite a good pedigree report. Therefore, such bulls are not found in certified or commercial semen stations for producing frozen semen doses. Hence, in the case of breeding bulls, unlike humans, low-concentrated or watery semen occasionally does not indicate pathological fertility problems. Thus, ejaculating watery semen may be a temporary condition due to insufficient sexual preparation and improper AV temperature during semen collection (15). Generally, the daily sperm production rate and ejaculate sperm concentration in different species highlight their genetic potential (16). The ejaculate concentration differed significantly between inter- and intra-bulls. The ejaculate sperm concentration of a particular buffalo is affected by a variety of environmental factors, including age, insufficient sexual preparation prior to sperm collection, environmental temperature, and the temperature of the artificial vagina (17). In the study, we observed 5,341 ejaculates of 31 buffalo bulls. We found that every buffalo bull donates ejaculates that have a sperm concentration of less than 500 million sperm/mL, which ranges from 1.33 to 34.33% of the ejaculates. The overall average of the ejaculates that had a sperm concentration of less than 500 million sperm/ mL was 9.0% in buffaloes. To know the percentage of buffalo ejaculates that meet the minimum qualifying criteria to store the semen doses for AI, the records of 5,565 buffalo semen ejaculates were examined. Based on 5,650 ejaculates, we are reporting for the first time that the ejaculates having a concentration between 300 and 400 million sperm/mL and >401- < 500 million/mL, 64.74 and 74.74% of total ejaculates met the minimum criteria, respectively. The maximum number of qualified ejaculates (77 to 81%) belonged to ejaculates whose concentration ranged from 500 to 1,400 million sperm/mL. The highest semen doses (61,767) (8.79%) were produced by the ejaculates that had a sperm concentration of between 1,101 and 1,201 million/mL. Thus, from the observations, it appears that ejaculate concentrations less than 500 million/mL also met the minimum qualifying criteria, cryopreserved and used for AI semen quality. As traditional microscopic-based quality control assessments alone may not provide a comprehensive evaluation of semen quality and the ability to predict bull fertility. Additional methodologies and technologies could enhance the accuracy and reliability of these assessments (18). Therefore, we decided to conduct a more in-depth study because it is not necessary for the ejaculate cryopreserved based on fresh and post-thaw sperm motility to qualify for other quality tests, indirectly affecting the conception rate. For this, we divided the ejaculates into four categories (<300, 300–400, >400 - < 500 and ≥ 500 million sperm/mL) based on ejaculate concentration. Therefore, after successful cryopreservation, the ejaculates were evaluated for membrane integrity, sperm abnormality, objective assessment of sperm motility, sperm kinematics, acrosomal intactness, mitochondrial membrane potential and superoxide production in frozen–thawed sperm. The functional integrity of the plasma membrane is of primary importance for the fertilizing abilities of a spermatozoon (19). The plasma membrane intactness of sperm of all four categories was evaluated by HOST because many times, sperm that have weak plasma membranes but under normal physiological conditions show normal sperm motility, but hypo-osmotic conditions make them vulnerable (20, 21). Comparatively, a higher number of sperm with intact plasma membranes was in category ≥500 than in category <300. The plasma membrane integrity of the other two categories (300–400 and >400- < 500) were intermediate of the two categories. The reported percentage of buffalo frozen–thawed spermatozoa with intact sperm membrane is 32.63% (21). The recommended minimum number of frozen–thawed sperm with intact sperm membrane is 40% (6). Considering the recommendation of a minimum of 40%, the ejaculates of categories < 300 are not suitable for the use of AI. Thus, the present study indicates that ejaculates of category <300 possess a weaker plasma membrane. The weakening or loss of the plasma membrane over the acrosome exposes the outer acrosomal membrane, resulting in the leaking of acrosomal enzymes (22). Hence, we evaluated the number of sperm with intact plasma membranes and intact acrosome. Rasul et al. (21) reported that about 62% of sperm had intact acrosomes after freeze–thaw in buffalo, but they did not distinguish between live and dead sperm with intact acrosomes. Because both dead and live sperm can have an intact acrosome. Similarly, the standard operative procedure (SOP) for government and commercial semen stations recommends 50% sperm with intact acrosomes. It is well established that up to 50% of spermatozoa die during the freeze–thaw process. Using the PI dye, we distinguished the sperm population into four categories: live sperm with intact acrosome, live sperm with damaged acrosome, dead sperm with intact acrosome and dead sperm with damaged acrosome. All three categories, except category <300, were 47 to 50% live sperm with intact acrosomes. The sperm mitochondria have been considered a double-edged sword (23, 24) because they produce ATP as well as excess free radicals once they are compromised. The mitochondrial membrane potential of spermatozoa drops after sperm cryopreservation, resulting in less ATP production and more superoxide anion production (24–26). The superoxide anion is the primary and main free radical produced by mitochondria (27). In frozen–thawed buffalo sperm, from 45 to 58.96% of the sperm subpopulation showed MitoSox-positive sperm in flow cytometry (9, 24). In the present study, only in category < 300, the MitoSox-positive sperm subpopulation was 64.20%, which was higher than early reports (9, 10, 24), and the other three categories fell within the range previously reported. The higher mitochondrial membrane potential (MMP) of sperm is required for ATP production. We found that except for the category <300, in all the other three categories, there was no difference in the percentage of spermatozoa with high MMP. The average value in the three categories was about 45.00% sperm with high MMP. Like our results, Arjun et al. (24) reported greater than 40% buffalo frozen–thawed sperm with high MMP from our laboratory. Similarly, Ahmed et al. (28) estimated the high MMP in frozen–thawed buffalo sperm, which was 44.85%. From these studies, it appears that the percentage of sperm with high MMP in the ejaculates of categories 300–400 and >400- < 500 were in a normal range. In our research, we found that highly low-concentrated ejaculates (< 300 million sperm/mL) are typically either rejected before/after cryopreservation or, if they meet minimum qualifying criteria based solely on post-thaw sperm motility, tend to fall short of meeting expected standards in other advanced quality assessment tests.

## 5 Conclusion

We meticulously examined the data from our laboratory over the last 5 years. We found that there was an average of 9% of buffalo ejaculates whose sperm concentration was less than 500 million sperm/ mL, and out of those, 48% of ejaculates whose concentration ranged from 300 to 500 million sperm/mL were qualified for the minimum criteria of the SOP guideline. Secondly, the qualified ejaculates from all four categories were analysed for several parameters by CASA and a flow cytometer. We observed that the ejaculates in the <300 category exhibited lower quality compared to others. The study suggests that ejaculates with a concentration exceeding 300 million sperm per mL may be suitable for cryopreservation and could potentially be utilized for artificial insemination (AI). However, it is important to note that further fertility data is needed to substantiate these findings more comprehensively.

## Data Availability

The raw data supporting the conclusions of this article will be made available by the authors, without undue reservation.
